# Unusual Good Functional Outcome After Surgical Management of Maluniting Schatzker Type II Fracture

**DOI:** 10.7759/cureus.11066

**Published:** 2020-10-20

**Authors:** Kishore Vellingiri, Hariprasad Seenappa, Satyarup Dasanna

**Affiliations:** 1 Orthopaedics, Sri Devaraj Urs Academy of Higher Education and Research, Kolar, IND

**Keywords:** maluniting schatzker’s fracture, foot drop

## Abstract

Tibial plateau fractures account for approximately 1% of all fractures. The reported incidence is about 10.3 per 100,000. Isolated tibial plateau fracture with articular step-off of 4 mm or less can be optimally treated with conservative management. An unstable joint requires further workup to determine whether fracture fragment movement or ligament pathology is the underlying cause of instability. We report the management of a case of delayed presentation of the proximal tibia with the neck of fibula fracture with foot drop.

## Introduction

Tibial plateau fractures account for approximately 1% of all fractures. The reported incidence is about 10.3 per 100,000 [1]. According to Wheeless, isolated tibial plateau fracture with articular step-off of 4 mm or less can be optimally treated with conservative management as long as the joint is stable. An unstable joint requires further workup to determine whether fracture fragment movement or ligament pathology is the underlying cause of instability [2]. Functional outcomes depend on early restoration of range of motion, joint stability, and adequate pain management [3]. We report a good functional outcome after surgical management of maluniting Schatzker fracture with foot drop.

The abstract of the case was presented at the ICOSMAS 2019 XIII International Conference on Orthopaedics, Sports Medicine and Arthroscopic Surgery in Bangkok, Thailand, December 17-18, 2019. The abstract is published in the online supplement of the abovementioned conference journal.

## Case presentation

A 31-year-old male patient with no known comorbidities was brought to our tertiary care center at Tamaka, Kolar, Karnataka, South India. The patient revealed a history of road traffic accident, due to which he sustained injury to the right lower limb. The patient had been unable to walk since then. He underwent native treatment for about six weeks and was brought to our hospital for further management. On examination, his vitals were stable. Local examination of the right leg showed swelling and tenderness present over the proximal third of the right leg. Range of motion at the right knee joint was 10-30 degrees. Assessment of range of motion at the ankle joint revealed that dorsiflexion was absent. Capillary refill time was normal. All other long bones and joints were found to be clinically normal.

**Figure 1:**
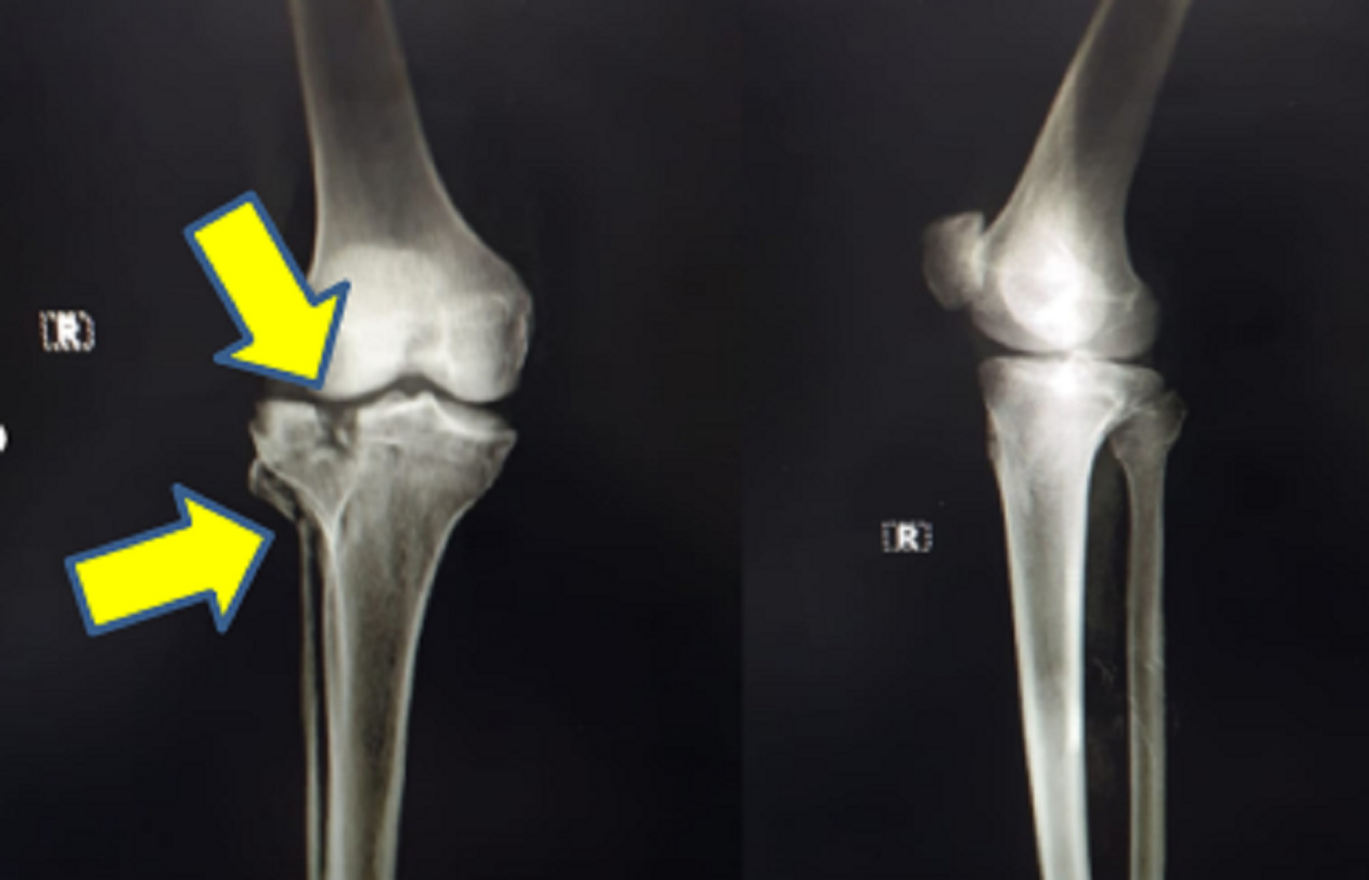
Plain radiograph (anteroposterior and lateral view) of the right knee showing split fracture involving the lateral tibial condyle, extending into the articular surface, and oblique fracture of the fibular head.

**Figure 2:**
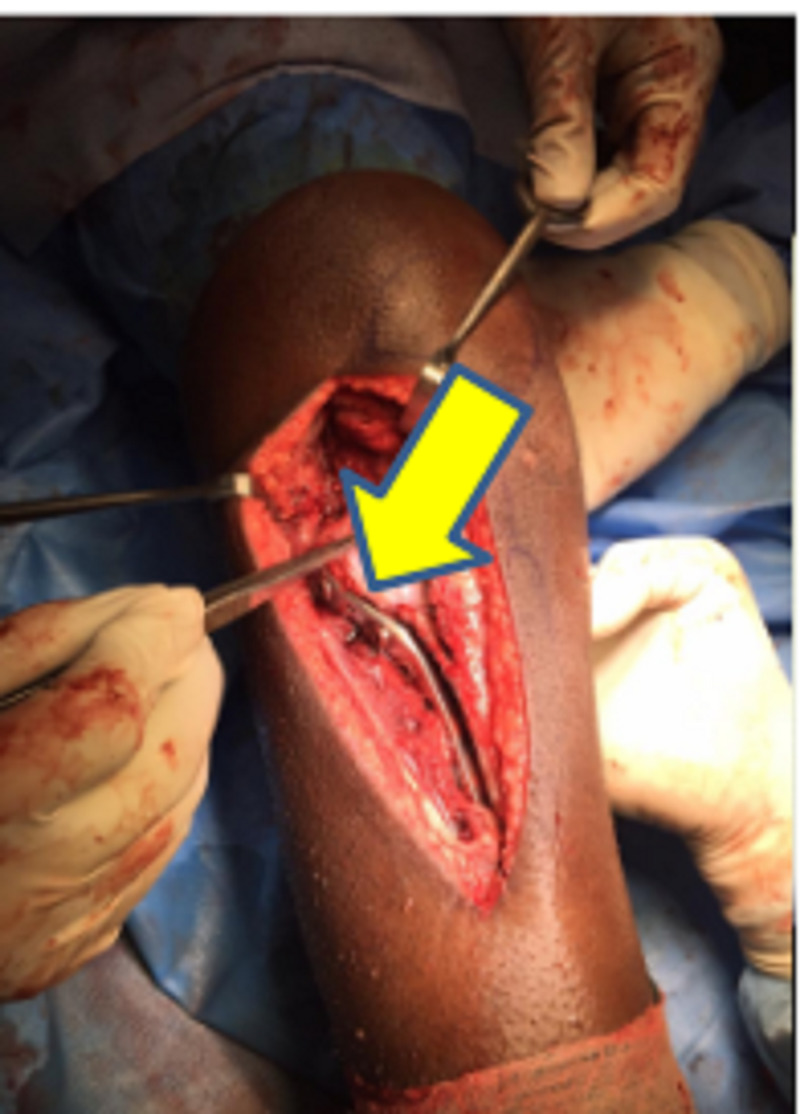
Intra-operative picture showing high-grade tear in the proximal aspect of the lateral collateral ligament and grade 2 medial collateral ligament tear, which was treated with open reduction and internal fixation with proximal tibia locking compression plating with autologous bone grafting from the ipsilateral iliac crest.

The patient was initially managed with intravenous administration of amoxicillin- potassium clavulanate 1.2 g prophylactically. The patient was then temporarily stabilized with an above-knee fiberglass splint. He was sent for preoperative imaging, as shown in Figure 1. Plain radiograph of the right knee showed split fracture involving the lateral tibial condyle extending into the articular surface, with lateral displacement of fracture fragments, and oblique fracture of fibular head with caudal displacement of fracture fragments. MRI of the right knee showed a high-grade tear in the proximal aspect of the lateral collateral ligament and a grade 2 medial collateral ligament tear. In addition, the patient was counselled regarding expectations and outcomes. After obtaining informed consent, the patient was operated under spinal anesthesia. Open reduction and internal fixation with proximal tibia locking compression plating with autologous bone grafting from the ipsilateral iliac crest (corticocancellous graft) along with lateral meniscectomy and arthrolysis of the knee joint was performed under spinal anesthesia, as shown in Figure 2. The post-operative period was uneventful.

Hospital course

**Figure 3:**
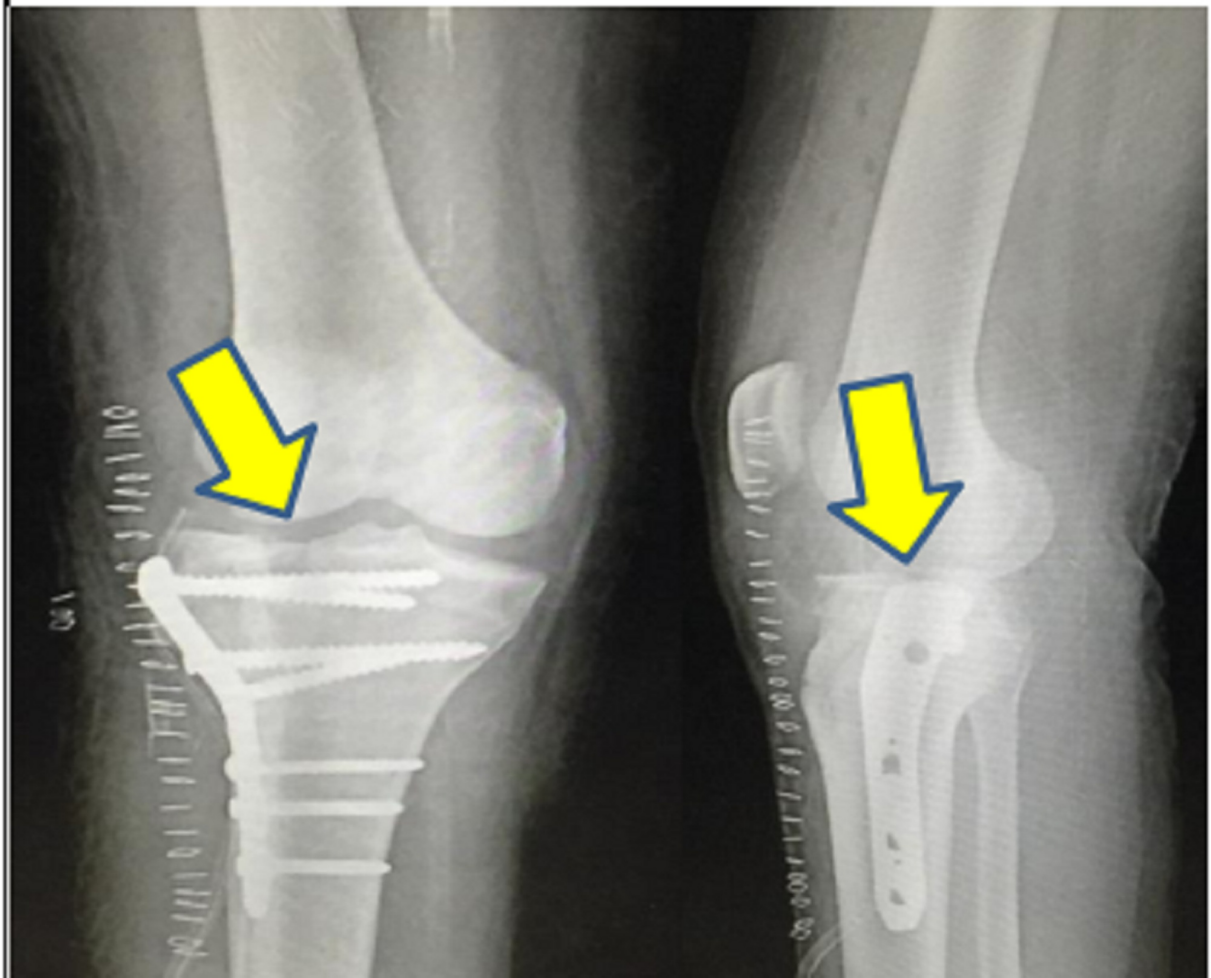
Plain radiograph (anteroposterior and lateral view) of the right knee showing reduced split fracture involving the lateral tibial condyle with locking compression plate.

Post-operatively, the patient received intravenous amoxicillin-potassium clavulanate 1.2 g twice daily for seven days and amikacin sulfate 500 mg twice daily for five days followed by oral amoxicillin-potassium clavulanate 625 mg twice daily for seven days. Post-operative radiograph of the operated knee joint was taken on post-operative day 2, as shown in Figure 3. An above-knee slab support was applied, and the patient was advised strict non-weight bearing for six weeks. Culture and sensitivity from surgical site showed staphylococcus epidermidis, which was sensitive to tetracycline and doxycycline. The patient was started on tablet doxycycline 100 mg twice a day for 14 days. All staples were removed on post-operative day 14, and the surgical site was found to be healthy. The patient underwent physiotherapy in the form of muscle stimulation test 15 minutes daily till he recovered from the foot drop. The guarded continuous passive motion was also started for the patient. At discharge, range of motion at the knee joint was 0- to 120-degree flexion after one month of physiotherapy. Tinel sign was positive 1-cm along the common peroneal nerve distribution at the level of the middle third of the leg. The patient had recovered from the foot drop (possibly due to neuropraxia). Partial weight bearing was initiated after six weeks. The patient was allowed full weight bearing at three months. At the last follow-up, six months post-injury, he was walking without any difficulty.

## Discussion

Tibia plateau fractures are complex intra-articular fractures. They can harbor concomitant soft tissue injuries. From a surgical perspective, these fractures are often difficult to treat. They have high complication rates, including premature onset of osteoarthritis related to incongruence of the healed joint [4]. Preoperative identification of meniscal injuries before any surgical intervention is desirable so as to enable the surgeons to contemplate a more prompt surgical strategy to repair these meniscal injuries. Approximately 55-70% of them affect the lateral plateau, 10-23% affect the medial plateau, and 10-30% affect both plateaus [5]. Schatzker type II fractures are the fracture patterns, which are most commonly encountered. It is defined as the depression of the articular surface of lateral tibia with sagittal or coronal split [6]. Schatzker type II fractures typically occur with forced valgus of the knee alone or in combination with axial compressive load during injury [7]. Ringus et al. [8] concluded that patients having more than or equal to 10 mm of lateral plateau diameter, as measured on CT images, had an eight-fold increase in risk of having a lateral meniscus tear. In another study, Gardner et al. [9] found that 6 mm of articular depression and 5 mm of condylar widening seen on plain radiographs were predictive of lateral meniscal injury in Schatzker type II tibial plateau fracture.

The meniscus plays a more critical role in a fractured tibial plateau. It acts as the roof of the damaged articular surface. Every effort should be made to preserve or repair the meniscus in tibial plateau fractures [5]. Foot drop may be due to a disturbance at any central or peripheral location along the motor neural pathway, which terminates in the dorsiflexor muscles of the foot, or at multiple locations in series. Optimal localization of the lesion is a pre-requisite for appropriate treatment and a successful outcome [10]. Any patient with a subjectively disturbing foot drop and a clinically suspected compressive neuropathy of the peroneal nerve must be informed about the choice of surgical decompression of the nerve at the fibular head. Many patients benefited from measures that are nonsurgical, including modification of activity, bracing, physical therapy, and medication. Surgical decompression should be reserved for refractory cases and those with compressive pattern, acute lacerations, or severe conduction changes [11].

In the posterolateral corner of the lateral plateau, there is a bare area that was unsupported by rafting screws. The conventional 3.5-mm locking compression plating was then pre-contoured. Thus, additional rim plating may be helpful in treating plateau fractures with a posterolateral fragment [12]. Definitive reduction and repair at 7-10 days post-injury have found surgical success, with fewer rates of infection [13]. Diabetes, hypertension, and pulmonary disease have a higher incidence of secondary problems. Smokers also have a higher chance of acquiring secondary problems. The delayed surgical protocol is intended to allow for the soft tissue healing so as to reduce rates of infection. Coupled with empiric intravenous antibiotics, infection rates could be still above 20% [14]. The optimal goal after a plateau fracture with associated repair/rehabilitation is a well-articulated joint with easy range of motion. Patients and providers ought to be mindful of any changes throughout the healing process, as prompt intervention may result in a more favorable outcome [15].

## Conclusions

Patients with delayed presentation of proximal tibia with neck of fibula fracture with foot drop may be managed successfully with proximal tibia locking compression plating with bone grafting, which may be autologous, and physiotherapy with better functional and radiological outcomes.

Our patient had presented after six weeks of native treatment with no improvement. This highlights the need for immediate surgical fixation of Schatzker type II fracture to prevent physical disability and mental anguish.

## References

[1] Elsoe R, Larsen P, Nielsen NPH, Swenne J, Rasmussen S, Osgaard SE (2015). Population-based epidemiology of tibial plateau fractures. Orthopedics.

[2] Wheeless C (2016). Tibial plateau fractures. Wheeless' Textbook of Orthopaedics.

[3] Timmers TK, Van der Ven DJC, De Vries LS, Van Olden GDJ (2014). Functional outcome of tibial plateau fracture osteosynthesis: a mean follow-up of six years. Knee.

[4] McNamara IR, Smith TO, Shepherd KL, Clark AB, Nielsen DM, Donell S, Hing CB (2015). Surgical fixation methods for tibial plateau fractures. Cochrane Database Syst Rev.

[5] Durakbasa MO, Kose O, Ermis MN, Demirtas A, Gunday S, Islam C (2013). Measurement of lateral plateau depression and lateral plateau widening in a Schatzker type II fracture can predict a lateral meniscal injury. Knee Surg Sports Traumatol Arthrosc.

[6] Schatzker J, McBroom R, Bruce D (1979). The tibial plateau fracture. The Toronto experience 1968-1975. Clin Orthop Relat Res.

[7] Schatzker J (2005). Fractures of the tibial plateau. The Rationale of Operative Fracture Care.

[8] Ringus VM, Lemley FR, Hubbard DF, Wearden S, Jones DL (2010). Lateral tibial plateau fracture depression as a predictor of lateral meniscus pathology. Orthopedics.

[9] Gardner MJ, Yacoubian S, Geller D (2005). The incidence of soft tissue injury in operative tibial plateau fractures: a magnetic resonance imaging analysis of 103 patients. J Orthop Trauma.

[10] Carolus AE, Becker M, Cuny J, Smektala R, Schmieder K, Brenke C (2019). The interdisciplinary management of foot drop. Dtsch Arztebl Int.

[11] Poage C, Roth C, Scott B (2016). Peroneal nerve palsy: evaluation and management. J Am Acad Orthop Surg.

[12] Kim Y, Yoon YC, Cho JW, Cho WT, Jeon NH, Oh CW, Oh JK (2018). Rim plate Augmentation of the posterolateral bare area of the tibial plateau using a 3.5-mm precontoured locking compression plate: a cadaveric study. J Orthop Trauma.

[13] Borrelli J (2014). Management of soft tissue injuries associated with tibial plateau fractures. J Knee Surg.

[14] Kokkalis ZT, Iliopoulos ID, Pantazis C, Panagiotopoulos E (2016). What’s new in the management of complex tibial plateau fractures?. Injury.

[15] Morris BJ, Unger RZ, Archer KR, Mathis SL, Perdue AM, Oremskey WT (2013). Risk factors of infection after ORIF of bicondylar tibial plateau fractures. J Orthop Trauma.

